# Identification and Experimental Validation of Prognostic miRNA Signature and Ferroptosis‐Related Key Genes in Cervical Squamous Cell Carcinoma

**DOI:** 10.1002/cam4.70415

**Published:** 2024-11-11

**Authors:** Yan Guo, Yana Han, Junjie Zhang, Yanbin Zhou, Meiyan Wei, Lijun Yu

**Affiliations:** ^1^ Department of Gynecology Shanxi Medical University First Hospital Taiyuan China; ^2^ Department of Neurosurgery Shanxi Medical University Second Hospital Taiyuan China; ^3^ Department of Teaching Affairs Section Shanxi Medical University First Hospital Taiyuan China

**Keywords:** cervical squamous cell carcinoma, ferroptosis‐related genes, miRNA signatures, prognostic biomarkers

## Abstract

**Objectives:**

This study aimed to investigate the prognostic value of miRNAs and ferroptosis‐related genes in cervical squamous cell carcinoma.

**Methods:**

We mined data from public databases for differentially expressed miRNAs, ferroptosis‐related genes, and clinical parameters and constructed a prognostic risk model. The predictive performance of the model was evaluated using survival and receiver operating characteristic curve analyses. We combined the clinicopathological features to construct a nomogram and evaluated its efficacy using calibration and clinical decision curves. The correlation between miRNA characteristics, risk score, and the tumor microenvironment was also studied. Next, consensus and key genes were screened, and their biological functions were analyzed using KEGG, GO, GSEA, and drug sensitivity analysis. Finally, the expression of miRNAs and key genes was detected using qRT‐PCR and western blotting to verify the prediction results.

**Results:**

Seven miRNA signatures (miR‐100‐3p, miR‐301a‐5p, miR‐331‐3p, miR‐425‐5p, miR‐502‐3p, miR‐505‐5p, and miR‐629‐3p) were generated, and prognostic risk and nomogram models were successfully constructed. These models exhibited good accuracy. miRNA signatures correlated with the tumor microenvironment. Twelve consensus genes and three key genes (SLC2A1, ANO6, and TXNIP) were screened and their biofunctional diversity was identified using various analytical methods. qRT‐PCR and western blotting were used to verify the expression of miR‐301a‐5p, miR‐505‐5p, SLC2A1, and TXNIP in cervical squamous carcinoma. The results were consistent with those of bioinformatics analyses.

**Conclusions:**

Seven miRNAs may serve as prognostic biomarkers of cervical squamous cell carcinoma. SLC2A1, ANO6, and TXNIP are associated with cervical squamous cell carcinoma and may serve as ferroptosis‐related markers of the disease.

## Graphical abstract

Seven miRNAs can serve as prognostic biomarkers for cervical squamous cell carcinoma. SLC2A1, ANO6, and TXNIP are associated with cervical squamous cell carcinoma and may be ferroptosis‐related markers of the disease.

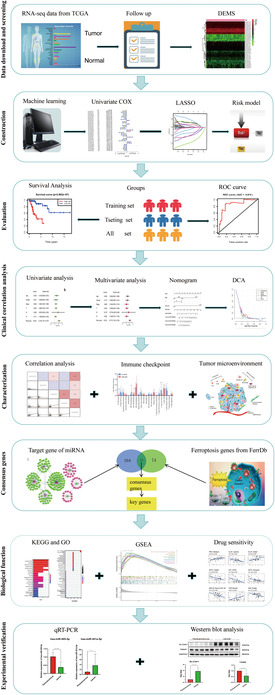

## 1 Introduction

According to data provided by the World Health Organization (WHO), global morbidity and mortality due to cervical cancer are increasing [1]. This is despite the implementation of cervical cancer screening programs, the advent of the HPV vaccine, and advances in cervical cytology and cervical pathology biopsy techniques, which have provided highly accurate methods for the detection of cervical cancer. However, its advanced manifestations, such as low survival and poor prognosis, make cervical cancer a pressing global public health concern [2]. Cervical cancer has two main subtypes: cervical squamous cell carcinoma (CESC) and adenocarcinoma. CESC accounts for approximately 75% of all cervical cancers. To date, the genetic mechanisms underlying cervical squamous carcinoma have not been fully elucidated, hindering the development of effective targeted therapeutic regimens. Therefore, identifying biomarkers with high sensitivity and specificity is important.

More than a decade ago, researchers discovered a new form of cell death [3] in 2012; Mr. Stockwell named it “ferroptosis.” This is a new mode of programmed cell death that differs from apoptosis and necrosis in terms of morphology, biochemistry, and genetics. It is mainly characterized by the iron‐dependent accumulation of lipid peroxidation, which manifests as an abnormal metabolism of intracellular lipid oxides catalyzed by excess iron ions, reactive oxygen species production, and polyunsaturated fatty acid overoxidation‐mediated regulated cell death [4]. Several studies have shown that ferroptosis plays an important role in physiological processes and diverse diseases [5], including T‐cell immunity [6], metabolic diseases [7], malignant tumors [8], neuronal injury disorders [9], ischemia perfusion disorders [10], and other diseases [11]. In addition to drug‐induced cell ferroptosis, several genes associated with tumor formation and progression have been identified as markers or modulators of ferroptosis. These ferroptosis‐related biomarkers are promising drug targets for the precise treatment of various cancers. However, there is a paucity of ferroptosis‐related biomarkers for early identification of cervical cancer.

MiRNAs are a group of non‐coding, single‐stranded RNA molecules that are approximately 22 nucleotides (nt) in length and encoded by endogenous genes [12], which can directly combine with the 3′‐untranslated region (3'‐UTR) of the target mRNA to regulate gene expression at the posttranslational level [13]. miRNAs regulate various biological processes such as apoptosis, proliferation, and metabolism [14] and play an important role in tumor development and prognosis [15]. Some studies have revealed that miRNA expression profiles promote cervical cancer progression and metastasis [16]. This indicates that miRNAs may serve as promising biomarkers for the diagnosis, prediction of survival, and precise treatment of cancers [17].

In this study, we identified and screened miRNAs with prognostic significance in cervical squamous carcinoma as well as key genes involved in ferroptosis using bioinformatics analyses. To validate the results of the bioinformatic analyses, we verified the expression of miRNAs and key genes using qRT‐PCR and western blotting. The screened prognostic miRNAs and key genes for ferroptosis can be used as biomarkers of cervical squamous cell carcinoma for future clinical treatment.

## 2 Materials and Methods

### 2.1 Data Preparation and Differential Expression Analysis

The data used in our study were mainly obtained from the TCGA database (https://portal.gdc.Cancer.gov/) [18], accessed February 10, 2023. We procured 254 specimens, encompassing 251 primary cervical squamous cell carcinoma patients and 3 healthy individuals. Their demographics are listed in Table S1. Notably, despite the disproportionate representation of cancer samples and normal specimens in this study, Zheng et al. [19] and Li et al. [20] in their antecedent research endeavors assessed the feasibility of deriving pertinent prognostic models replete with diagnostic markers and clinically salient therapeutic targets from comparably imbalanced sample cohorts. Differentially expressed miRNAs (DEMs) were screened using EdgeR, gplot, and limma R language packages. The screening standards used were |log2FC| > 1 and Padj < 0.05.

Ferroptosis‐related genes, including driver, suppressor, and marker genes, were sourced from the Ferroptosis‐related Database (FerrDB) (http://www.zhounan.org/ferrdb/index.html) [21], visited on February 10, 2023. Differential analysis was used to screen differentially expressed genes from ferroptosis‐related genes, and the resulting DEG was identified. The DEG will be intersected with the differential miRNA target genes in subsequent studies, so that the resulting intersected genes are both differential miRNA target genes and differential ferroptosis‐related genes. The screening standard was |log_2_FC| > 1 and *P*_adj_ < 0.05.

### 2.2 Construction of a Prognostic Risk Model

All samples with complete information were randomly assigned to either the training group (*n* = 121) or the test group (*n* = 121). In the training group, miRNAs associated with prognosis were screened using univariate Cox regression analysis. The miRNA signatures were further screened using LASSO and multivariate Cox regression analyses. Using the results of multivariate Cox analysis, the risk score was calculated by the following formula: risk score = *β*1 × expression (miRNA1) + *β*2 × expression (miRNA2) + … + *βn* × expression (miRNAn). Subsequently, the predictive values for patients with cervical squamous cell carcinoma in the training set, test set, and all the sets were calculated. Finally, a prognostic risk model was constructed.

### 2.3 Evaluation of Prognostic Risk Model

After establishing a prognostic risk model, it must be thoroughly evaluated. First, the patient samples were grouped into low‐ and high‐risk groups based on their risk scores. Subsequently, Kaplan–Meier survival curves were used to examine the differences in survival between the groups. Next, risk score curves were plotted to illustrate the differences in risk scores according to the model. Survival plots were constructed to determine the survival status of each sample. Heat maps were plotted to show differences in the expression levels of prognostic miRNAs between the low‐ and high‐risk groups. We evaluated the predictive ability of the prognostic model using time‐dependent receiver operating characteristic (ROC) curves. Finally, to elucidate the relationship between prognosis and risk scores, as well as clinical characteristics (age, stage, and grading), we used univariate and multivariate Cox regression analyses, and the results were presented in the form of forest samples.

### 2.4 Construction and Valuation of Nomogram

Risk scores and clinical factors, including age, tumor stage, and grade, were analyzed using univariate Cox regression analysis to screen for factors significantly related to survival (*p* < 0.1). Multivariate Cox regression analysis was used to identify candidate predictors that were significantly related to survival (*p* < 0.05). Nomograms were constructed using these predictors, and scores in the nomogram model were assigned to these variables. The total score for each patient was obtained by adding the scores of predictors filled in the nomogram model. Finally, the patients' survival outcomes at 1, 3, and 5 years were calculated using the total score and probability of survival outcome. Comparison of clinicopathological characteristics and nomogram prediction ability using time ROC analysis to estimate the discrimination and accuracy of the nomogram model. Decision curve analysis (DCA) was performed to evaluate clinical utility.

### 2.5 Tumor Microenvironment Analysis

The tumor immune microenvironment is closely related to the therapeutic effects and prognosis of patients with malignant tumors. Therefore, we examined the relationship between miRNA signatures and immune cells in cervical squamous cell carcinoma. The CIBERSORT algorithm by R was used to determine the relationship between the risk score and the abundance of 21 tumor‐infiltrating immune cells in cervical squamous cell carcinoma samples. Stromal scores, immune scores, and tumor purity were calculated using the ESTIMATE algorithm by R package. Based on the Spearman test, the correlation between the miRNA signature and the infiltration level of 21 immune cells was analyzed. Immune checkpoint molecules, including inhibitory and stimulatory molecules, are defined as ligand–receptor pairs that exert inhibitory or stimulatory effects on immune responses. We compared the expression of the 46 most common immune checkpoint genes (CD274, CD86, CTLA4, HAVCR2, LAG3, PDCD1, TIGIT, CD200, and LA1R1) between the high‐ and low‐risk groups using the LIMMA R package.

### 2.6 Prediction Target Gene and Screening Consensus Genes and Key Genes

To ascertain the target genes associated with the seven miRNAs, we harnessed online analytical instruments, specifically TargetScan [22], miRDB [23], and miRBase [24] to prognosticate potential candidates. The confluence of miRNA target genes from the three databases was meticulously discerned using a Venn diagram. Subsequently, an overlaying procedure was performed wherein differentially expressed ferroptosis‐related genes were juxtaposed with the aforementioned target genes. The intricate interplay between miRNAs and their target genes was elucidated using Cytoscape software [25]. In this study, Kaplan–Meier survival analysis was used to further explore the relationship between the 12 consensus genes and the survival of patients with cervical squamous cell carcinoma. Genes significantly related to OS were screened and named key genes.

### 2.7 Exploration of the Biological Functions of Differentially Expressed Genes and Key Genes

To gain a deeper understanding of the biological relevance inherent to differentially expressed ferroptosis‐related genes, we used DAVID to perform enrichment analysis of the KEGG pathways (http://www.kegg.jp/) [26] and GO functional annotations (http://geneontology.org/) [27]. GO functional annotations encompass biological processes (BP), cellular components (CC), and molecular functions (MF). To clarify the potential regulatory mechanisms of SLC2A1 and TXNIP in cervical squamous cell carcinoma, we conducted a single‐gene Gene Set Enrichment Analysis (GSEA) to identify pivotal KEGG signaling pathways and GO functional attributes.

### 2.8 Drug Sensitivity Analysis

The CellMiner database (https://discover.nci.nih.gov/cellminer/home.do) was used to download drug sensitivity data. Next, we combined the consensus gene expression data with drug sensitivity data to perform the Pearson correlation test. Finally, the correlation between consensus gene expression and drug sensitivity was determined. Predictive pharmaceutical agents predominantly include chemotherapeutic agents and targeted therapeutics, which are widely employed in clinical practice. Regulatory network mapping was based on the results of the regulatory relationship between miRNAs and their target genes, and the correlation between consensus genes and drugs using Cytoscape.

### 2.9 Clinical Tissue Collection and Ethics Statement

From January 2023 to June 2023, we procured a total of 10 specimens comprising cervical squamous cell carcinoma tissues and their adjacent paracancerous tissues from the Shanxi Medical University First Hospital. The selection criteria for inclusion in this investigation encompassed the following prerequisites: (1) a conclusive diagnosis of cervical squamous cell carcinoma rendered by a certified pathologist, and subsequent histopathological assessment and staging conducted in accordance with WHO standards; (2) surgical resection of tumor lesions; and (3) no history of antecedent chemotherapy, radiotherapy, or other therapeutic interventions for malignancies prior to the surgical procedures. The research protocol was approved by the Ethics Committee of Shanxi Medical University First Hospital (Ethical number: KYLL‐2023‐060). All samples were obtained with written informed consent and analyzed anonymously.

### 2.10 Quantitative Real‐Time Polymerase Chain Reaction (qRT‐PCR)

Initially, we isolated total RNA from the tissue specimens using a FastPure Cell/Tissue Total RNA Isolation Kit (TaKaRa, 9109). Subsequently, we synthesized miRNA first‐strand cDNA (via the stem loop) (Vazyme, MR101‐01) to effectuate the conversion of RNA into complementary DNA (cDNA). Following this pivotal step, we amplified cDNA using the miRNA Universal SYBR PCR Master Mix (MQ101‐01; Vazyme) in conjunction with the ABI 7900HT FAST real‐time system. We meticulously computed the relative expression levels of each miRNA, with small nuclear RNA U6 serving as the internal reference standard. The primer sequences used in this study are listed in Table 1.

**TABLE 1:** Sequences of primers used in the present research.

Primer	Sequences (5' to 3')
hsa‐miR‐505‐5p‐RT	GTCGTATCCAGTGCAGGGTCCGAGGTATTCGCACTGGATACGACACATCA
hsa‐miR‐505‐5p‐F	GCGGGGAGCCAGGAAGTAT
hsa‐miR‐505‐5p‐R	AGTGCAGGGTCCGAGGTATT
hsa‐miR‐301a‐5p‐RT	GTCGTATCCAGTGCAGGGTCCGAGGTATTCGCACTGGATACGACAGTAGT
hsa‐miR‐301a‐5p‐F	CGCGGCTCTGACTTTATTGC
hsa‐miR‐301a‐5p‐R	AGTGCAGGGTCCGAGGTATT
RNU6‐1‐F	CTCGCTTCGGCAGCACA
RNU6‐1‐R	AACGCTTCACGAATTTGCGT

### 2.11 Western Blot Analysis

In the initial phase of our study, RIPA lysis buffer (Beyotime, Shanghai, China, P0013B) was used to facilitate protein extraction from cervical cancer tissue specimens. Subsequently, protein concentration was quantified using the BCA method (Beyotime, Shanghai, China, P0011). Proteins were separated using SDS‐PAGE (Beyotime, Shanghai, China, P0523S), followed by their transfer onto a polyvinylidene fluoride membrane (Beyotime, Shanghai, China, FFP33). Thereafter, the membranes were incubated with primary antibodies directed against SLC2A1 (1:500, ZENBIO, 380464), TXNIP (1:500, Abcam, ab188865), and GAPDH (1:10,000, Abcam, ab8245) overnight. Following this, a meticulous tripartite washing regimen was undertaken, using Western Wash Buffer (Beyotime, Shanghai, China, P0023C) on each occasion, with each washing cycle spanning 10 min. Subsequently, the corresponding enzyme‐conjugated secondary antibodies (1:2000; Proteintech, SA00001) were incubated for 1 h. Finally, protein bands were visualized. It is important to note that GAPDH served as the internal control in this experimental paradigm.

## 3 Results

### 3.1 Differentially Expressed miRNAs and Genes

**FIGURE 1:**
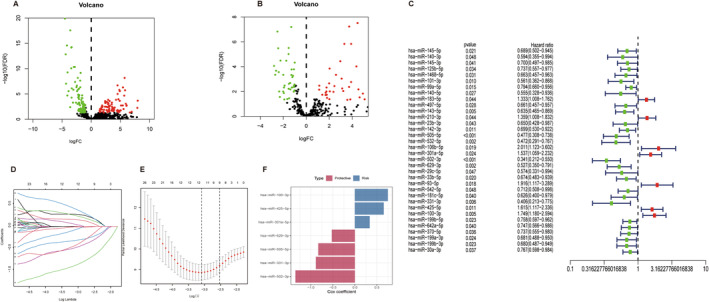
Differentially expressed miRNAs and ferroptosis‐related genes, and miRNA‐based prognostic signature in CESC. (A) Volcano plot of DEMs. (B) Volcano plot of DEGs. (C) Forest plot of univariate Cox analysis of DEMs filtered 34 miRNAs. (D) Evaluation of the change in risk (HR trajectory) using Lasso regression. (E) Survival cross‐validated partial log‐likelihood deviance for assessment of the fit of the Cox model. (F) Expression bar graph for 7 miRNA features.

Here, we explored DEMs based on the TCGA data set along with the clinical information of these patients, including age, staging, and grading information. The 191 differentially expressed miRNAs were identified based on the criteria of FDR < 0.05 and |log_2_FC| > 1.0. Differentially expressed miRNAs were identified by comparing cancer and normal tissues. Meanwhile, 86 differentially expressed iron pendant‐associated genes (DEGs) were identified using *P*_adj_ < 0.05 and |log_2_FC| > 1 as screening criteria. Volcano plots showed significant differences in the distribution of foldchanges in DEMs (Figure 1A) and DEGs (Figure 1B).

### 3.2 Construction of Seven miRNA‐Based Signature for Prognostic Risk Model

All eligible patients were randomly allocated to either the training or testing cohort. In the training cohort, our univariate Cox regression analysis uncovered 34 miRNAs with prognostic relevance through univariate Cox regression analysis, as elegantly depicted in Figure 1C. Subsequently, seven miRNAs (miR‐100‐3p, miR‐301a‐5p, miR‐331‐3p, miR‐425‐5p, miR‐502‐3p, miR‐505‐5p, and miR‐629‐3p) were identified using a combination of multivariate Cox regression and LASSO analyses, as showcased in Figure 1D,E. Notably, although five of these seven miRNAs have been previously associated with cervical cancer in the existing literature, miR‐502‐3p and miR‐301a‐5p have not been previously reported to be involved in cervical cancer. Among these seven miRNAs, we observed the upregulated expression of miR‐301a‐5p, miR‐331‐3p, miR‐425‐5p, and miR‐629‐3p, whereas the remaining three exhibited downregulated expression profiles. Formulation of the prognostic model comprising these seven miRNAs was meticulously achieved through multivariate Cox analysis (as delineated in Table 2), and the expression of the seven miRNA features is shown as a bar graph in Figure 1F. The model's mathematical representation is succinctly articulated as follows: Risk score = (0.75 × expression of miR‐100‐3p) + (0.34 × expression of miR‐301a‐5p) + (−0.89 × expression of miR‐331‐3p) + (0.66 × expression of miR‐425‐5p) + (−1.35 × expression of miR‐502‐3p) + (−0.83 × expression of miR‐505‐5p) + (−0.52 × expression of miR‐629‐3p).

**TABLE 2:** Multivarirate analysis of cervical squamous cell carcinoma patients.

miR	Coefficient
hsa‐miR‐505‐5p	−0.830798265
hsa‐miR‐502‐3p	−1.350031516
hsa‐miR‐629‐3p	−0.524543505
hsa‐miR‐331‐3p	−0.885943263
hsa‐miR‐425‐5p	0.656067681
hsa‐miR‐100‐3p	0.749338745
hsa‐miR‐301a‐5p	0.336711484

### 3.3 Evaluation of Prognostic Risk Model

**FIGURE 2:**
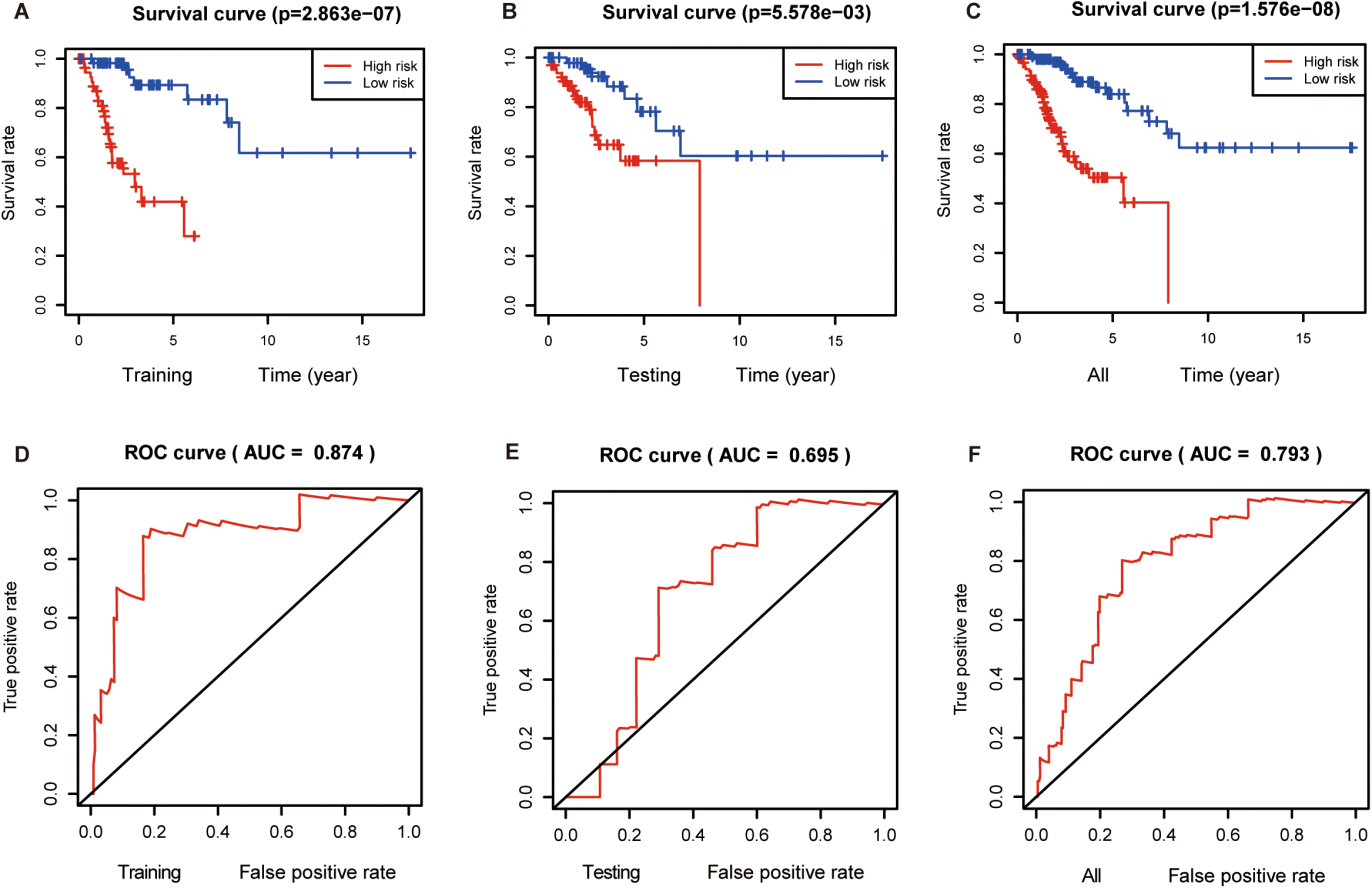
Validation of the prognostic signature. The Kaplan–Meier curves, the ROC curve between the low‐ and high‐risk patients of three groups. (A) The Kaplan–Meier curves of the training set. (B) The Kaplan–Meier curves of testing set. (C) The Kaplan–Meier curves of all set. (D) The ROC curve in the training set. (E) The ROC curve in the testing set. (F) The ROC curve in all sets.

**FIGURE 3:**
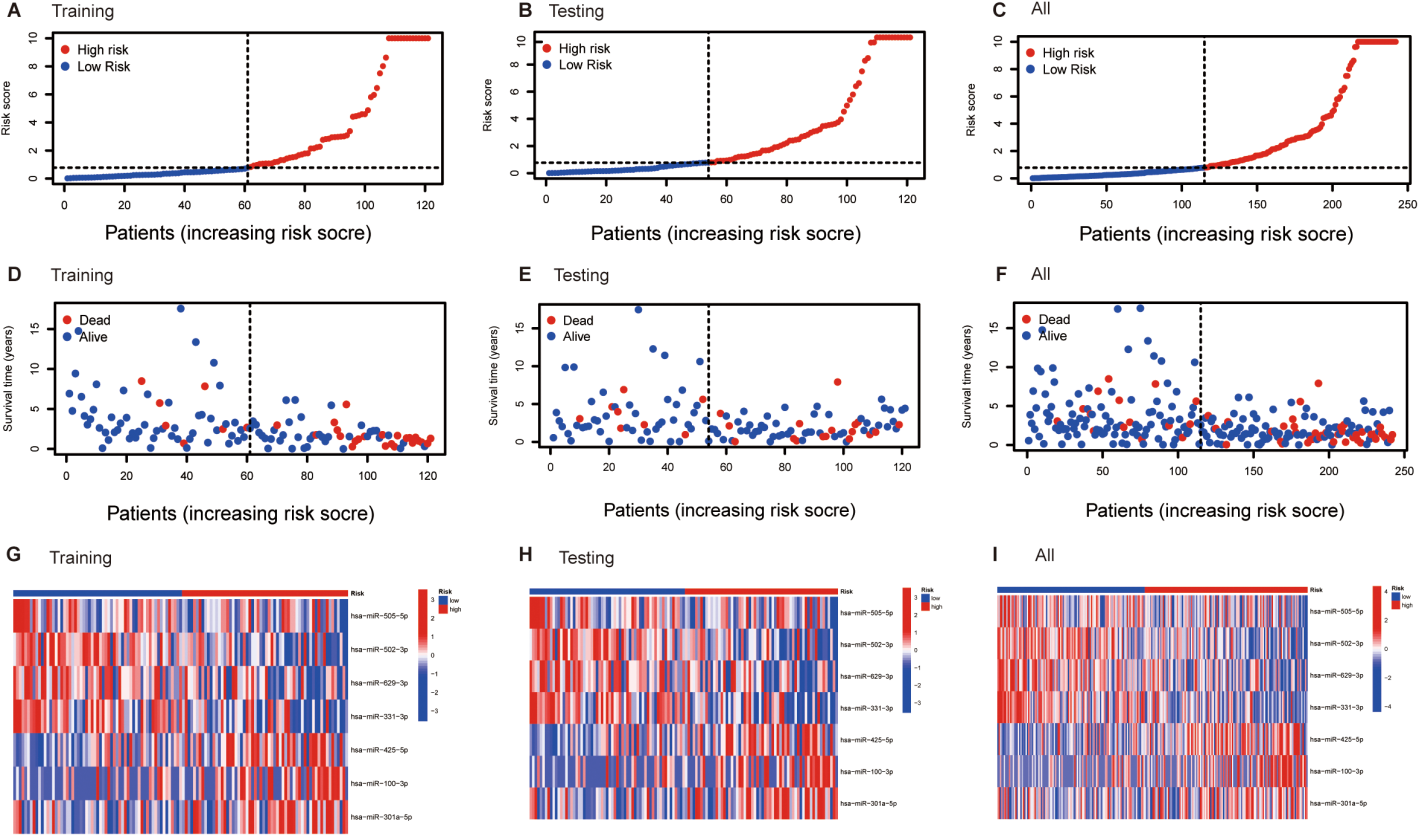
The risk score curve, the survival status map, and the expression heatmap between the low‐ and high‐risk patients of three groups. (A) The risk score curve between the low‐ and high‐risk patients in the training set. (B) The risk score curve in the testing set. (C) The risk score curve in all sets. (D) The survival status map between the low‐ and high‐risk patients in the training set. (E) The survival status map in the testing set. (F) The survival status map in all sets. (G) The expression heatmap between the low‐ and high‐risk patients in the training set. (H) The expression heatmap in the testing set. (I) The expression heatmap in all sets.

**FIGURE 4:**
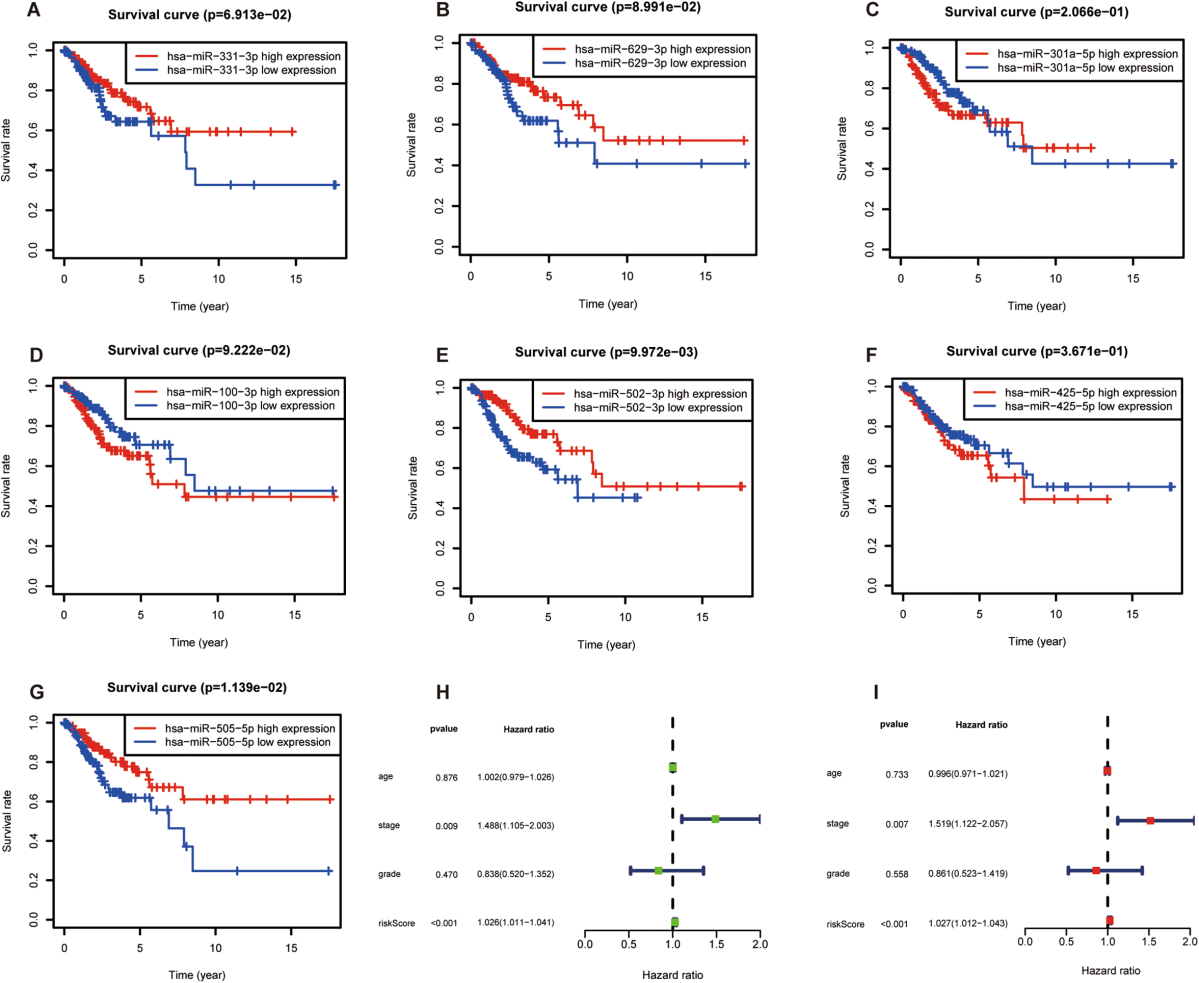
Differential expression analyses of the prognostic signature with clinicopathological characteristics in CESC patients. (A–G) The Kaplan–Meier curves of seven miRNAs associated with OS of patients. (H) Forest plot of univariate regression analysis. (I) Forest plot of multivariate Cox regression analysis.

Following the formulation of the prognostic risk model, a rigorous evaluation was performed. First, Kaplan–Meier survival analyses were conducted separately for the training, testing, and combined sets. The Kaplan–Meier survival analysis plot for the training set graphically illustrated a lower overall survival rate in the high‐risk group, revealing a higher survival rate in the low‐risk group (Figure 2A). Analogous and noteworthy findings were corroborated by both the testing and combined data sets (Figure 2B,C). Moreover, the risk score curve underscored the discernible distinction between the low‐ and high‐risk groups, with the former displaying a lower risk score and the latter a higher risk score (Figure 3A–C). The survival state diagram effectively portrayed that the low‐risk group had an extended survival duration, in stark contrast to the high‐risk group, which was characterized by a higher mortality rate (Figure 3D–F). The heatmap in Figure 3G–I unmistakably depicted a direct relationship between low miRNA expression levels and high‐risk scores, whereas high miRNA expression levels were inversely correlated with risk scores. The assessment of the predictive efficacy of our established cervical squamous cell carcinoma prognosis risk model was further supported by the ROC curves. The AUC for the three distinct groups were 0.874, 0.695, and 0.793, respectively (Figure 2D–F). These AUC values confirmed the suitability of our model for assessing the survival risk of patients with cervical squamous cell carcinoma, thereby substantiating its predictive capabilities. Consequently, this prognostic risk model has the potential to be a prognostic tool for future clinical applications. Differential expression analysis of the prognostic characteristics, OS, and clinicopathological characteristics of patients was then carried out. A Kaplan–Meier curve revealed the prognostic relevance of these seven miRNAs in patients with cervical squamous cell carcinoma. Among these, miR‐100‐3p, miR‐301a‐5p, and miR‐425‐5p demonstrated negative correlations, whereas the remaining four miRNAs exhibited positive correlations (Figure 4A–G). Subsequently, the results derived from univariate and multivariate Cox regression analyses revealed that the risk score and stage functioned as independent risk factors for cervical squamous cell carcinoma(Figure 4H,I).

### 3.4 Construction and Valuation of Nomogram

**FIGURE 5:**
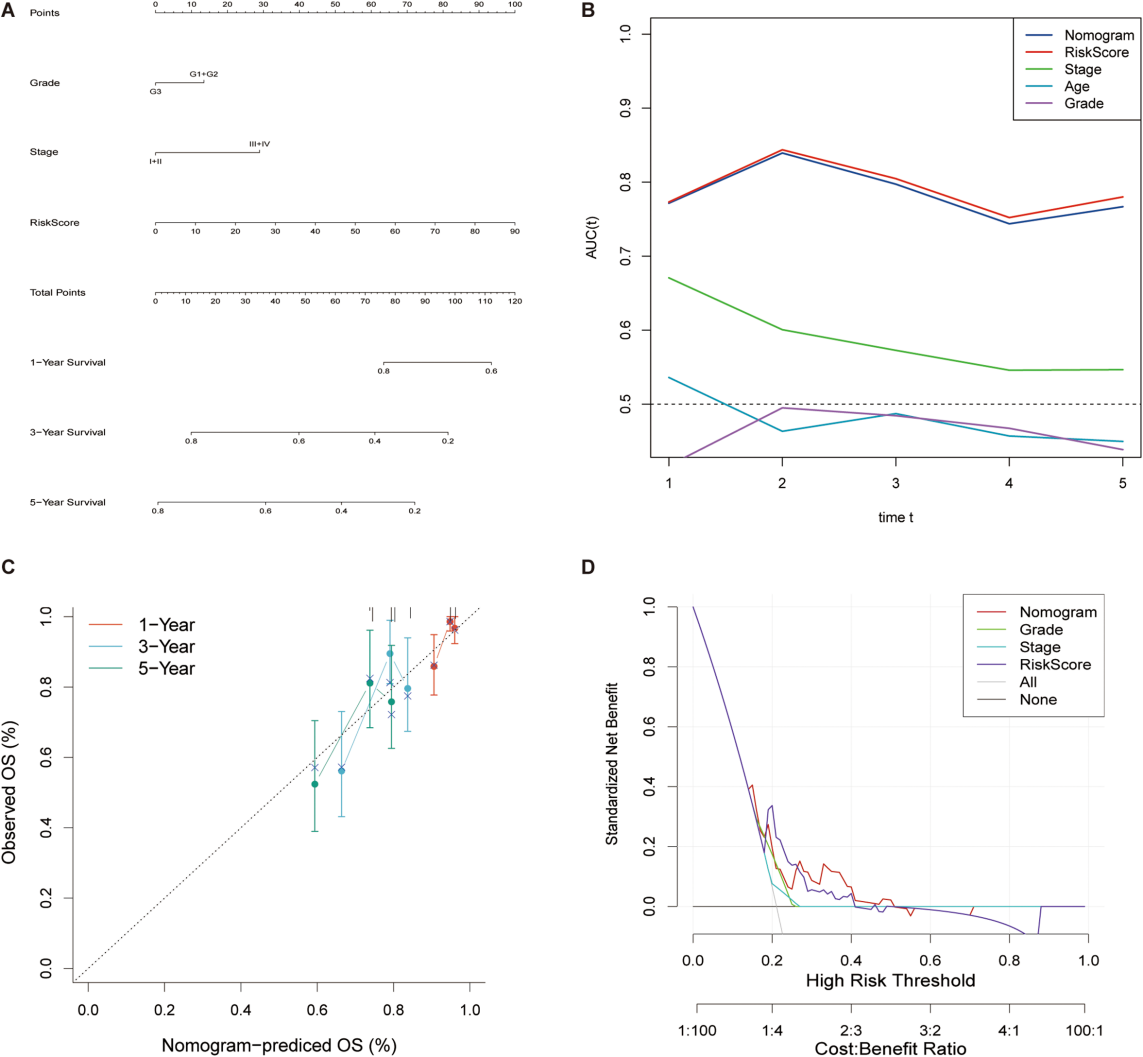
Nomogram and its evaluation. (A) Nomogram. (B) Time ROC analysis. (C) The calibration curves. (D) The DCA curves.

We integrated the risk score and important clinical features such as Grade and Stage and constructed a nomogram (Figure 5A) that can be used to quantitatively predict the prognosis of patients and provide a reference for clinical decision‐making. As shown in the figure, those with tissue differentiation grade G3 were regarded as the reference and given 0 points, whereas those with G1 or G2 were given 14 points. Similarly, stage I or II was 0, whereas stage III or IV was 30. The score for each patient was calculated as the sum of the weight integrals for each variable. Time ROC analysis showed that the risk score and AUC of Nomogram in the TCGA cohort were higher than those of other indicators. ROC curves demonstrated good discrimination (Figure 5B). The calibration curves of the OS nomogram model at 1, 3, and 5 years also showed that the predicted results were in good agreement with the actual observational results (Figure 5C). Compared with a single clinical feature, DCA showed that the Nomogram model presented the highest benefit in predicting the prognosis of cervical squamous cell carcinoma (Figure 5D). The nomogram predicted more benefits than either the intervention—all or intervention—none strategies at different threshold points.

### 3.5 Tumor Microenvironment Analysis

**FIGURE 6:**
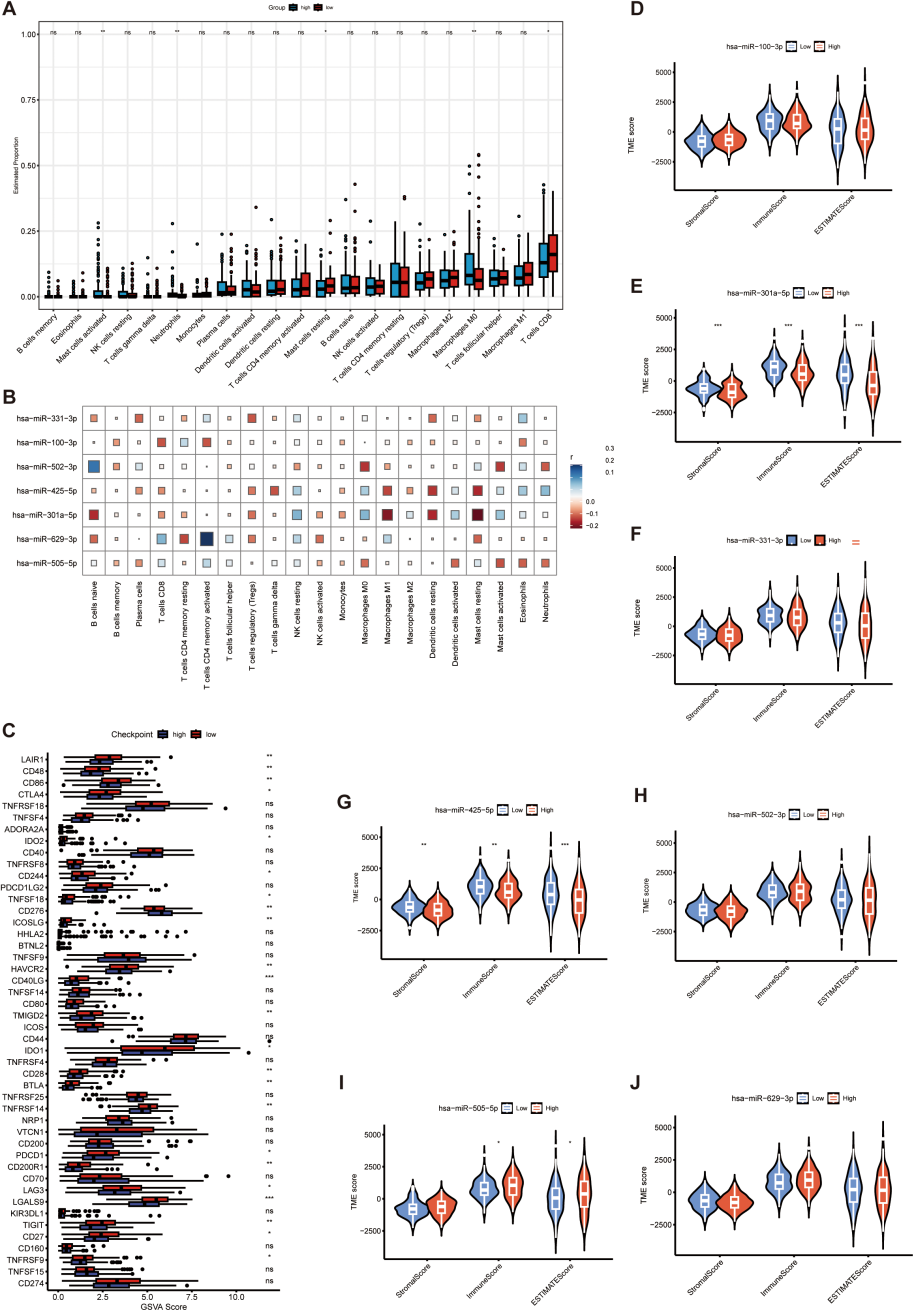
Analysis of tumor microenvironment and immune checkpoints analysis. (A) Immune infiltration patterns between high‐ and low‐risk patients. The abscissa and the ordinate represent the type and proportion of immune cells. (B) Correlation heat map of the relationship between miRNA signature and 21 immune cells. Blue indicates a positive correlation, and red indicates a negative correlation. The darker the color, the bigger the square, indicating the greater the correlation. Cells with a correlation close to 0 are basically colorless. (C) Analysis of immune checkpoints in two risk groups. (D–J) Correlation analysis of seven miRNAs signatures with immune score, matrix score, and comprehensive score.

As shown in the immunoinfiltration graph (Figure 6A), the levels of activated mast cells (*p* < 0.01), neutrophils (*p* < 0.01), and macrophages M0 (*p* < 0.01) were remarkably higher in the high‐risk group. In contrast, the numbers of resting mast cells (*p* < 0.05) and CD8+ cells (*p* < 0.05) were higher in the low‐risk group. Figure 6B shows the correlation analysis between miRNA and 21 immune cells, and we observe that miR‐301a‐5p was positively correlated with dendritic cell activation, mast cell activation, M0 macrophages, and NK cell resting. miR‐502‐3p positively correlated with naïve B cells and negatively correlated with neutrophils, activated mast cells, and macrophages M0. miR‐505‐5p was negatively correlated with eosinophil, and mast cell activation miR‐629‐3p positively correlated with resting NK cells, M1, T‐cell CD8, and T‐cell CD4 memory activated. From Figure 6D–J, we observed that the expression of miR‐301a‐5p and miR‐425‐5p was negatively correlated with the immune, matrix, and comprehensive scores (*p* < 0.01). The expression of miR‐505‐5p was positively correlated with the immune, matrix, and comprehensive scores (*p* < 0.05). We compared the expression of 46 common immune checkpoint genes between the two risk groups. As shown in Figure 6C, the expression levels of immune checkpoints in almost all high‐risk groups were significantly lower than those in the low‐risk groups. Combined with these results, we found that different risk groups of patients with cervical squamous cell carcinoma could accurately indicate the immune level; that is, the overall immune response of patients in the high‐risk group was lower than that of patients in the low‐risk group.

### 3.6 Prediction Target Gene and Screen Consensus Genes and Key Genes

**FIGURE 7:**
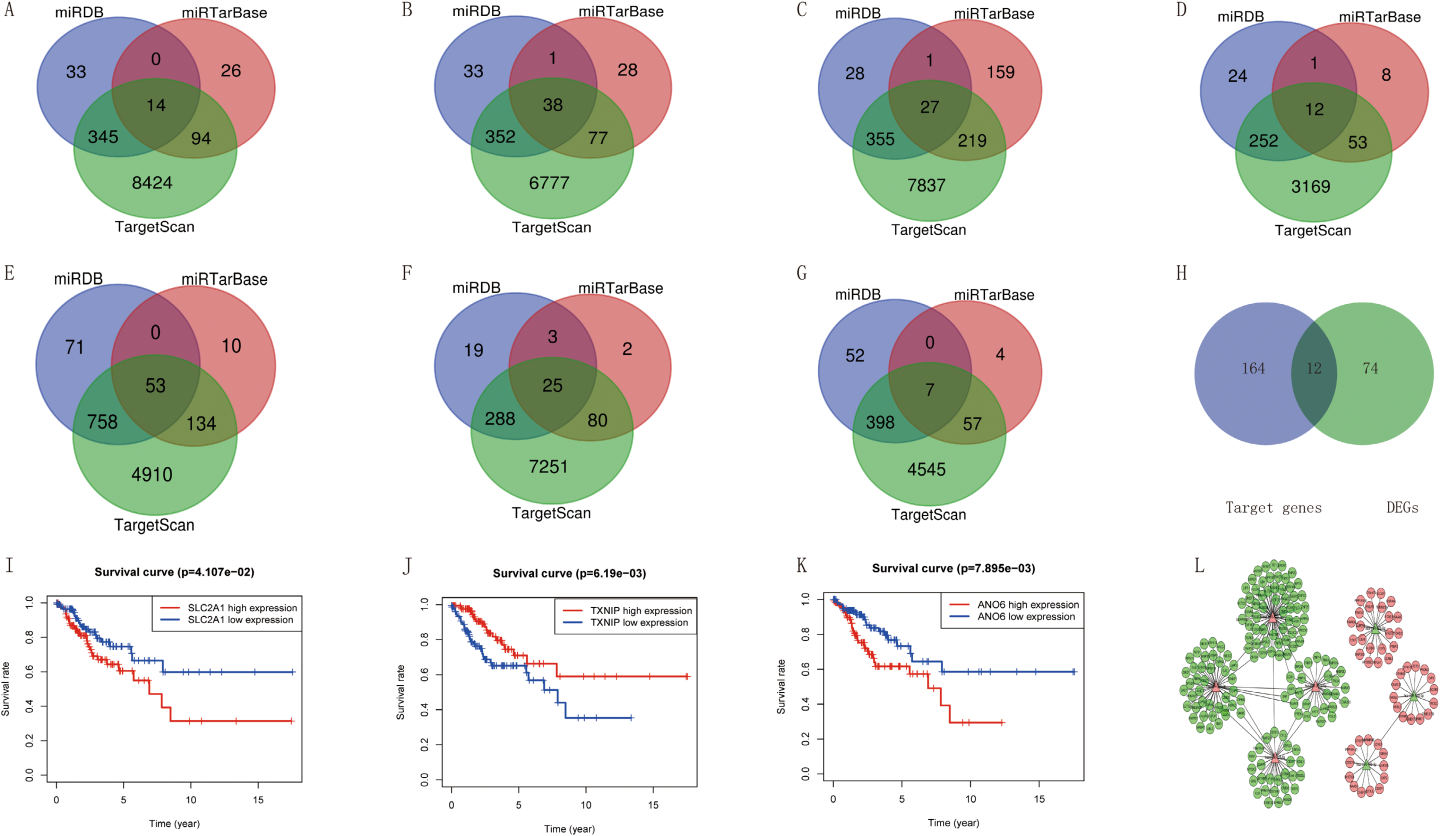
Target genes for seven miRNAs and consensus genes and key genes in CESC. (A) miR‐505‐5p, (B) miR‐425‐5p, (C) miR‐331‐3p, (D) miR‐502‐3P, (E) miR‐629‐3p, (F) miR‐100‐3p, (G) miR‐301a‐5p. (H) The overlap between 86 DEGs and 176 target genes. (I) The association of SLC2A1 with patients' prognosis of cervical cancer. (J) The association of TXNIP with patients' prognosis of cervical cancer. (K) The association of ANO6 with patients' prognosis of cervical cancer. (L) Network shows the intricate interplay between miRNAs and their target genes. Red denotes upregulated; green denotes downregulated.

To predict the target genes of the seven miRNAs, we used three online analysis tools: TargetScan, miRDB, and miRBase. The resultant Venn diagram showcased the presence of 14, 38, 27, 12, 53, 25, and 7 overlapping target genes across the three databases for miR‐505‐5p, miR‐425‐5p, miR‐331‐3p, miR‐502‐3P, miR‐629‐3p, miR‐100‐3p, and miR‐301a‐5p, respectively (Figure 7A–G). This comprehensive analysis unveiled a grand total of 176 target genes associated with the seven prognostic miRNAs. Intriguingly, among the 86 differentially expressed genes (DEGs) and 176 target genes, a select group of 12 consensus genes emerged (PROM2, SLC2A3, SLC2A1, PTEN, LIFR, EGR1, ACADSB, WWTR1, ANO6, TXNIP, NRAS, and PRKCA), indicating their significance in cervical cancer (Figure 7H and Table 3). To gain a deeper insight into the intricate interplay between miRNAs and their target genes, we constructed a regulatory network diagram, as shown in Figure 7L. It is discernible from this illustration that miR‐301a‐5p, miR‐425‐5p, and other miRNAs were upregulated, whereas miR‐505‐5p and miR‐502‐3p were downregulated. Notably, TXNIP was identified as a target gene of miR‐301a‐5p, and a negative regulatory relationship was identified between them. Similarly, SLC2A1 was identified as a target gene of miR‐505‐5p, and a negative regulatory interaction was identified between them. This study further examined the 12 consensus genes using Kaplan–Meier survival analysis. The results of this analysis underscored the significant prognostic relevance of ANO6, SLC2A1, and TXNIP (Figure 7I–K). Consequently, these three genes were identified as key genes. Interestingly, TXNIP exhibited a positive correlation with the survival rate and prognosis of patients with cervical squamous cell carcinoma, whereas ANO6 and SLC2A1 showed the opposite trend; both genes were negatively correlated with the survival rate and prognosis of patients. The expression of the three key genes in patients with cervical squamous carcinoma and in normal subjects is shown inFigure 8E. SLC2A1 is highly expressed in cervical squamous carcinoma tissues (*p* < 0.05). AN06 is lowly expressed in cervical squamous carcinoma tissues (*p* < 0.01), and similarly TXNIP is lowly expressed in cervical squamous carcinoma tissues compared to mormal tissues (*p* < 0.001).

**TABLE 3:** 12 consensus genes.

Symbol	logFC
PROM2	3.126272544
SLC2A3	−1.941961349
SLC2A1	3.725385032
PTEN	−1.076815944
LIFR	−3.73037064
EGR1	−2.393501513
ACADSB	−1.388468889
WWTR1	−1.510407702
ANO6	−1.531037498
TXNIP	−2.508846388
NRAS	1.133277141
PRKCA	−2.24737904

**FIGURE 8:**
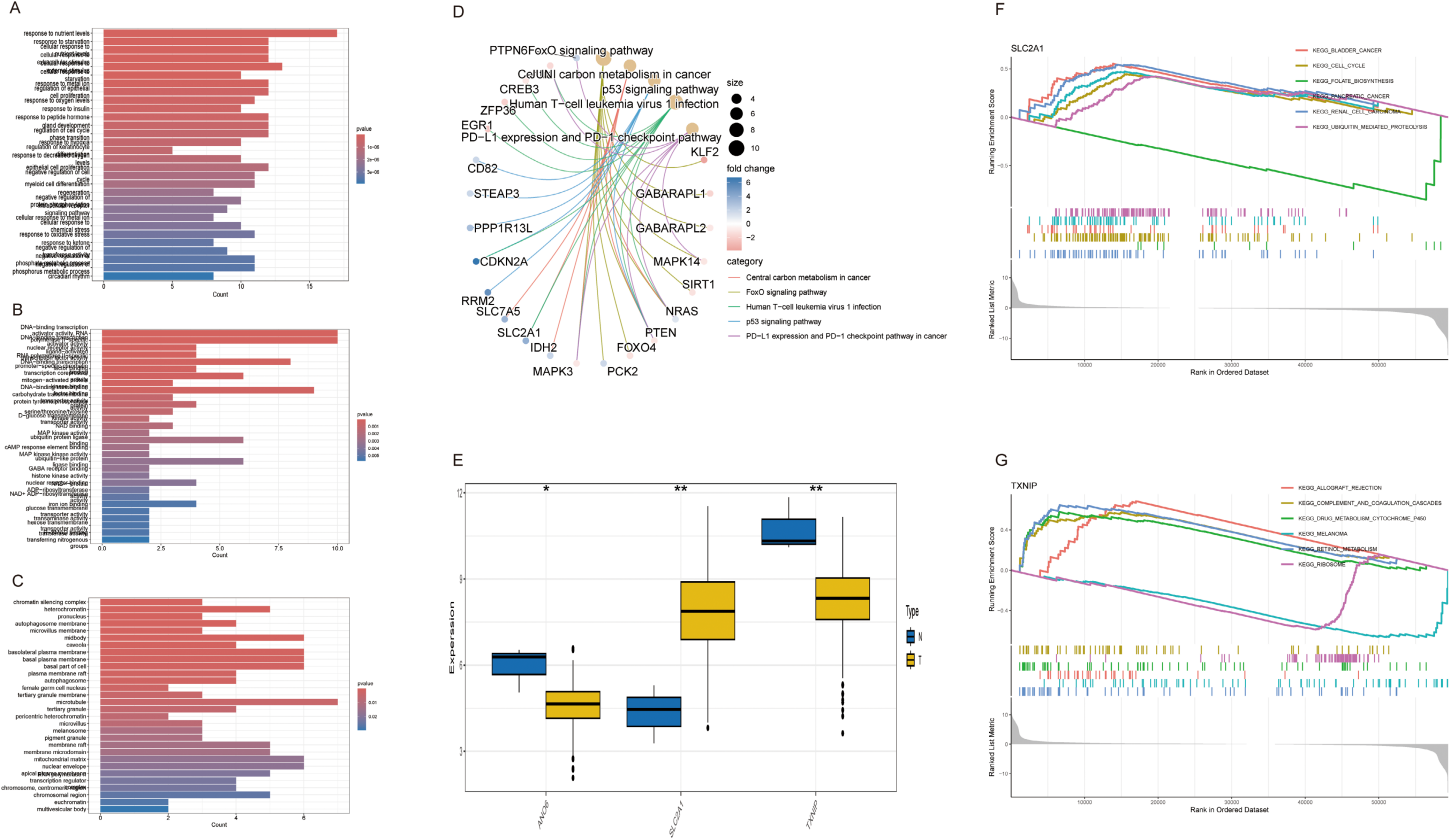
Biological functions of differentially expressed genes and key genes. (A) Bar plots showing GO analysis for BP. (B) Bar plots showing GO analysis for MF. (C) Bar plots showing GO analysis for CC. (D) Circos plots of KEGG analysis. (E) Box plots of expression levels of key genes. *p < 0.05, **p < 0.01, ****p < 0.001. (F) The single‐gene GSEA analysis of SLC2A1. (G) The single‐gene GSEA analysis of TXNIP.

### 3.7 Exploration of the Biological Functions of Differentially Expressed Genes and Key Genes

We imported 86 differentially expressed ferroptosis genes for the enrichment analysis. The outcomes of the GO analysis are shown in Figure 8A–C. In the realm of biological process (BP) GO annotations, we found a spectrum of vital processes, including responses to nutrient levels, starvation, and cellular responses to extracellular stimuli. Notably, the response to nutrient levels as the BP annotation had the most profound significance, as evidenced by its remarkably low *p* value of 2.04E‐11. In the molecular function (MF) GO annotations, the enrichment pathways in the top three positions were DNA‐binding transcription activator activity, RNA polymerase II‐specific activity, DNA‐binding transcription activator activity, and nuclear receptor activity. Within the expanse of cellular component (CC) GO annotations, we obtained enrichments, including the chromatin silencing complex, chromatin, pronucleus, and autophagosome membranes. Among these, the chromatin–silencing complex was the most enriched. Furthermore, the top five important enriched signaling pathways identified by KEGG analysis were the FoxO signaling pathway, central carbon metabolism in cancer, p53 signaling pathway, Human T‐cell leukemia virus 1 infection, PD‐L1 expression, and PD‐1 checkpoint pathway in cancer (Figure 8D). Among these, the FoxO signaling pathway exhibits the most profound significance and is important in physiology, pathology, and disease treatment. Figure 8F,G shows the single‐gene GSEA analysis of SLC2A1 and TXNIP, we observed that SLC2A1 is mainly concentrated in KEGG bladder cancer, cell cycle, pancreatic cancer, renal cell carcinoma, ubiquitin‐mediated proteolysis pathway, and it is upregulated. TXNIP is mainly involved in allograft rejection, complement and coagulation cascades, drug metabolism, cytochrome p450, and the retinol metabolism pathway. SLC2A1 and TXNIP were enriched in many important pathways. Deep insights into these signaling pathways may reveal the potential mechanisms involved in tumor development.

### 3.8 Drug Sensitivity Analysis

**FIGURE 9:**
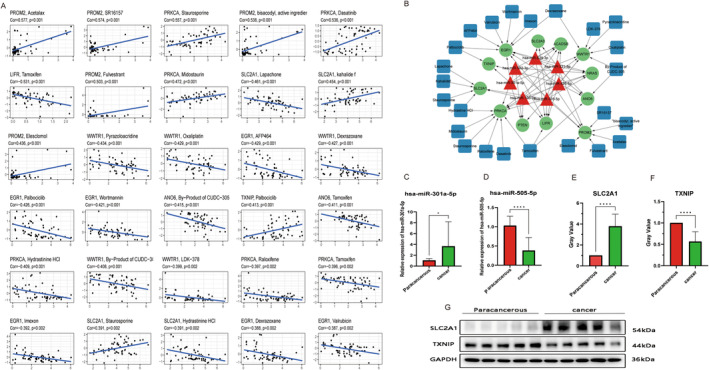
Drug sensitivity and experimental verification of miRNA signature and key genes. (A) Scatter diagram of the relationship between consensus genes and drug sensitivity. (B) Network diagram of miRNA and target gene and drug correlation. (C) Differential expression of miR‐301a‐5p between cervical squamous cell carcinoma tumor and paracancerous tissues. (D) Differential expression of miR‐505‐5p between cervical squamous cell carcinoma tumor and paracancerous tissues. (E) SLC2A1 expression in cervical cancer and paracancerous tissues. (F) TXNIP expression in cervical cancer and paracancerous tissues. (G) The Stripe plots of Western Blotting analysis. *p < 0.05, **p < 0.01, ****p < 0.001.

From the analysis of consensus gene and drug sensitivity (Figure 9A), we got that the expression of PROM2 was positively correlated with the sensitivity of Acetalax, SR16157, bisacodyl, active ingredient, Fulvestrant and Elesclomol (*p* < 0.001). The expression of SLC2A1 was positively correlated with sensitivity to kahalide and staurosporine (*p* < 0.001) but negatively correlated with sensitivity to lapachone and hydrazine (*p* < 0.001). The higher the expression of ANO6, LIFR, and PRKCA, the weaker the sensitivity to tamoxifen (*p* < 0.001). The drug sensitivity to palbociclib was positively correlated with the expression of TXNIP but negatively correlated with the expression of EGR1 (*p* < 0.001). The findings of the drug sensitivity analysis showed that the consensus genes had good reactivity to many common chemotherapeutic drugs and targeted drugs in patients with cervical squamous cell carcinoma. Network relationships were mapped using the regulatory relationships between miRNAs and target genes as well as the results of consensus genes and drug‐sensitivity correlations (Figure 9B). miRNA‐target gene relationships are derived from Figure 7L, and drug‐consensus gene relationships are derived from Figure 9A, where miRNAs are shown in Table 2 and consensus genes are shown in Table 3. The complex relationship between miRNAs and consensus genes and drugs can be seen in the network diagram, drugs and miRNAs regulate target genes, making an indirect association between miRNAs and drugs, and visualization making these associations clearer.

### 3.9 Quantitative Real‐Time Polymerase Chain Reaction (qRT‐PCR)

In this study, we procured a set of ten meticulously paired fresh cervical squamous cell carcinoma tissues and their corresponding paracancerous counterparts for qRT‐PCR analysis. This precise experimental endeavor revolved around the investigation of two specific miRNAs. The outcomes revealed a conspicuous contrast in the expression patterns of these miRNAs. Specifically, the expression level of miR‐301a‐5p was notably elevated in cervical squamous cell carcinoma tissues, whereas a concomitant reduction was observed in para‐cancerous tissues (*p* < 0.05). Concurrently, the expression of miR‐505‐5p was decreased in cervical squamous cell carcinoma tissues, whereas it was elevated in paracancerous tissues (*p* < 0.001). These compelling findings are graphically depicted in Figure 9C,D, consistently with the conclusions derived from our intricate bioinformatics analyses.

### 3.10 Western Blot Analysis

In our quest for a comprehensive understanding of key gene expression in cervical squamous cell carcinoma and corresponding paracancerous tissues, we performed western blot analyses. In this meticulously designed experiment, we focused on two pivotal genes: SLC2A1 and TXNIP. As shown in Figure 9E–G, a striking disparity was observed when comparing the expression levels in tumor tissues with those in their paracancerous counterparts. Specifically, the expression of SLC2A1 in tumor tissues exhibited a remarkable increase, whereas the expression of TXNIP demonstrated a noteworthy decrease. These empirical observations are consistent with the results of our rigorous bioinformatic analyses.

## 4 Discussion

Cervical squamous carcinoma remains a threat to human health. microRNAs are involved in the regulation of ferroptosis genes in a variety of cancers and nonmalignant diseases. However, the role of ferroptosis in cervical squamous cell carcinoma remains unclear. Therefore, an in‐depth study of miRNAs and ferroptosis associated with cervical squamous cell carcinoma is important. In this study, using bioinformatics analysis, we screened prognostic miRNAs and key ferroptosis genes related to cervical squamous cell carcinoma. Seven miRNA signatures (miR‐100‐3p, miR‐301a‐5p, miR‐331‐3p, miR‐425‐5p, miR‐502‐3p, miR‐505‐5p, and miR‐629‐3p) were generated, and prognostic risk and nomogram models were successfully constructed. Three key genes (SLC2A1, ANO6, and TXNIP) were screened and their biofunctional diversity was identified using various analytical methods. qRT‐PCR and western blotting were used to verify the expression of miR‐301a‐5p, miR‐505‐5p, SLC2A1, and TXNIP in cervical squamous carcinoma. The results were consistent with those of bioinformatics analyses.

Research has shown that different miRNAs can be used as biomarkers for multiple types of diseases, such as cancers, viral infections, cardiovascular disorders, etc. miR‐301a‐5p plays the role of a proliferating gene in cancer and cell development. It has been reported to be dysregulated in a variety of malignant tumors. For example, miR‐301a has been described as a potential marker of metastasis in prostate cancer and its high expression is associated with an increased risk of recurrence [28]. miR‐301a overexpression has also been observed in gastric cancer [29] and breast cancer [30].

Increasing evidence indicates that the aberrant expression of miR‐505‐5p plays a significant role in carcinogenesis. A previous study showed that the downregulation of miR‐505‐5p in breast tumor tissues is one of the most valuable noninvasive biomarkers for distinguishing patients with breast cancer at an early stage [31]. Another study has demonstrated that miR‐505‐5p is dysregulated in the cancer tissues of patients [32].

Based on our predictions, we identified three key genes that were significantly correlated with ferroptosis and cervical squamous cell carcinoma. Specifically, SLC2A1 and ANO6 were upregulated, whereas TXNIP was downregulated. In the present study, we found that TXNIP is one of the target genes of miR‐301a‐5p and that a negative regulatory relationship exists between them. SLC2A1 is the target gene of miR‐505‐5p, and the relationship between them is negatively regulated. ANO6 is a target gene of miR‐331‐3p, and there was a negative regulation between them. These three key genes are involved in ferroptosis in cervical squamous cell carcinoma.

Glucose can meet the energy demands of malignant cell proliferation [33]. An important characteristic of cancer cell metabolism is heightened glycolysis, which results in increased transport of glucose across cell membranes [34]. Glucose transporter promotes glucose uptake through the plasma membrane. Among the many glucose transporter family members, one such transporter is solute transporter family 2 member 1 (SLC2A1), which is also called GLUT1 [35]. It is expressed in many human cells, including cancer and normal cells [36]. Researchers have found that SLC2A1 promotes glycolysis in tumor cells, thus promoting their proliferation, invasion, and migration of tumor cells [37]. Previous studies have consistently shown aberrant expression of SLC2A1 in numerous cancers and that it is related to poor prognosis. SLC2A1 expression demonstrated prognostic significance. For instance, Liu et al. identified SLC2A1 as a biomarker of immune infiltration in colorectal cancer [38]. Min et al. showed that SLC2A1 can inhibit CD8+ T cells and B cells, which can improve the survival rate of patients with gastric cancer, and found that the upregulation of SLC2A1 has a statistically significant difference in DFS/DSS [39]. In our study, western blotting results showed that SLC2A1 was upregulated in cervical squamous cell carcinoma. Many tools, such as Kaplan–Meier and Cox regression analyses, have been used to confirm that the expression of SLC2A1 is related to the prognosis of patients with cervical squamous cell carcinoma. The survival rate of patients with high SLC2A1 expression was lower and vice versa. Therefore, high SLC2A1 expression may be considered a risk factor for cervical squamous cell carcinoma. This finding was similar to that reported by Reyna‐Her et al. [40]. These results indicate the importance of SLC2A1 in cervical squamous cell carcinoma.

Thioredoxin‐interacting protein (TXNIP) is a multifunctional protein and a key regulatory factor in the redox cleaning system. TXNIP mediates oxidative stress, inhibits cell enhancement, and induces apoptosis by inhibiting the function of thioredoxin system [41]. TXNIP is considered to be involved in tumor suppression by negatively regulating the function of the TRX system, increasing oxidative stress, and regulating immune cells. Thus, TXNIP expression is downregulated in many tumors, such as breast [42], lung [43], hepatocarcinoma [44], and prostate cancer [45]. In this study, western blot analysis results confirmed that TXNIP expression is downregulated in cervical squamous cell carcinoma tissues. Kaplan–Meier analysis showed that the prognosis of patients with cervical squamous cell carcinoma is better than that of patients with downregulated TXNIP. Therefore, TXNIP is an anticancer factor in cervical squamous cell carcinoma. We hope that this method can be used for the diagnosis and treatment of cervical squamous cell carcinoma in the future.

In summary, the miRNAs and key genes screened in this study play important roles in various diseases, especially malignant tumors. Some of these miRNAs and key genes have been identified in a study of cervical squamous carcinoma; however, some have not been reported. We hope that these findings provide a reference for the study of cervical squamous cell carcinoma. In this study, because of the single research method, limited sample size, lack of external database validation, and budgetary constraints, we only screened prognostic miRNA signatures and key ferroptosis‐related genes associated with cervical squamous carcinoma and failed to clarify the interrelationship between them. Our common wish is to further explore the regulatory relationships and mechanisms of these miRNAs and key genes in subsequent studies and hopefully contribute to the research on cervical squamous cell carcinoma.

## 5 Conclusions

Our study showed that seven miRNAs (miR‐100‐3p, miR‐301a‐5p, miR‐331‐3p, miR‐425‐5p, miR‐502‐3p, miR‐505‐5p, and miR‐629‐3p) can serve as prognostic biomarkers for cervical squamous cell carcinoma. Meanwhile, SLC2A1, ANO6, and TXNIP are associated with cervical squamous cell carcinoma and may be ferroptosis‐related markers of this disease.

## Author Contributions

**Yan Guo:** investigation (equal), resources (equal), writing – original draft (equal). **Yana Han:** writing – review and editing (equal). **Junjie Zhang:** data curation (equal), formal analysis (equal), software (equal). **Yanbin Zhou:** visualization (equal). **Meiyan Wei:** validation (equal). **Lijun Yu:** conceptualization (equal), methodology (equal).

## Ethics Statement

This research protocol received the ethical imprimatur of the Ethics Committee at the Shanxi Medical University First Hospital (Ethical number: KYLL‐2023‐060). All the samples were obtained with written informed consent and analyzed anonymously.

## Conflicts of Interest

The authors declare no conflicts of interest.

## Supporting information


**Table S1.** The demography of the primary cervical cancer samples.

## Data Availability

Publicly available data sets were analyzed in this study. The data sets TCGA and the corresponding clinical patient information analyzed for this study can be found in the TCGA Knowledge Base (https://portal.gdc.cancer.gov, accessed on February 10, 2023). Ferroptosis‐related information analyzed for this study can be found in FerrDB (http://www.zhounan.org/ferrdb/index.html, accessed on February 10, 2023).
