# Supplementary material for: Identification and Experimental Validation of Prognostic miRNA Signature and Ferroptosis‐Related Key Genes in Cervical Squamous Cell Carcinoma
Source: Cancer Med. 2024 Nov 11;13(21):e70415. doi: 10.1002/cam4.70415 (PMC11551785; doi:10.1002/cam4.70415)
Supplement: Supplementary file 1 — Table S1. The demography of the primary cervical cancer samples. [file CAM4-13-e70415-s001.doc]

| Parameters | TCGA（N=251） | clinical（N=10） |
| --- | --- | --- |
| Age |  |  |
| ≤41,n（%) | 80（31.9） | 2（20.0） |
| ＞41,n（%） | 171（68.1） | 8（80.0） |
| Gender |  |  |
| Female,n（%） | 251（100.0） | 10（100.0） |
| Male,n（%） | — | — |
| Histology |  |  |
| squamous cell carcinoma,n（%） | 251（100.0） | 10（100.0） |
| adenocarcinoma,n（%） | — | — |
| other types,n（%） | — | — |
| Stage |  |  |
| FIGO IA-B,n（%） | 142（56.6） | 6（60.0） |
| FIGO IIA-B,n（%） | 58（23.1） | 4（40.0） |
| FIGO IIIA-C,n（%） | 32（12.7） | — |
| FIGO IVA-B,n（%） | 19（7.6） | — |
| TNM |  |  |
| T1,n（%） | 138（54.9） | 6（60.0） |
| T2,n（%） | 70（27.9） | 4（40.0） |
| T3,n（%） | 19（7.6） |  |
| T4,n（%） | 11（4.4） |  |
| TX,n（%） | 13（5.2） |  |
| N0,n（%） | 170（67.7） | 8（80.0） |
| N1,n（%） | 32（12.7） | 2（20.0） |
| Nx，n（%） | 49（19.6） |  |
| M0,n（%） | 109(43.4) |  |
| M1,n（%） | 11(4.4) |  |
| Mx,n（%） | 131(52.2) |  |
| Grade |  |  |
| G1,n（%） | 20（8.0） | 1(10.0) |
| G2,n（%） | 109（43.4） | 4(40.0) |
| G3,n（%） | 99（39.4） | 1(10.0) |
| GX,n（%） | 23（9.2） | 4(40.0) |
| Survival state |  |  |
| live,n（%） | 191（76.1） | 10（100.0） |
| dead,n（%） | 60（23.9） | — |

Table S1 The demography of the primary cervical cancer samples.
